# Interleukin-1 Receptor Antagonist as Therapy for Traumatic Brain Injury

**DOI:** 10.1007/s13311-023-01421-0

**Published:** 2023-08-23

**Authors:** Caroline Lindblad, Elham Rostami, Adel Helmy

**Affiliations:** 1 Department of Medical Sciences, Uppsala University, Uppsala, Sweden; 2 Department of Neurosurgery, Uppsala University Hospital, entrance 85 floor 2, Akademiska Sjukhuset, 751 85 Uppsala, Sweden; 3 Department of Clinical Neuroscience, Karolinska Institutet, Stockholm, Sweden; 4 Department of Neuroscience, Karolinska Institutet, Stockholm, Sweden; 5 Division of Neurosurgery, Department of Clinical Neurosciences, University of Cambridge, Cambridge, UK

**Keywords:** Traumatic brain injury, Neuroinflammation, Interleukin-1, Interleukin-1 receptor antagonist, Anakinra, Randomized controlled clinical trial, Personalized medicine, Secondary insult, Neurocritical care, Neurotrauma

## Abstract

Traumatic brain injury is a common type of acquired brain injury of varying severity carrying potentially deleterious consequences for the afflicted individuals, families, and society. Following the initial, traumatically induced insult, cellular injury processes ensue. These are believed to be amenable to treatment. Among such injuries, neuroinflammation has gained interest and has become a specific focus for both experimental and clinical researchers. Neuroinflammation is elicited almost immediately following trauma, and extend for a long time, possibly for years, after the primary injury. In the acute phase, the inflammatory response is characterized by innate mechanisms such as the activation of microglia which among else mediates cytokine production. Among the earliest cytokines to emerge are the interleukin- (IL-) 1 family members, comprising, for example, the agonist IL-1β and its competitive antagonist, IL-1 receptor antagonist (IL-1ra). Because of its early emergence following trauma and its increased concentrations also after human TBI, IL-1 has been hypothesized to be a tractable treatment target following TBI. Ample experimental data supports this, and demonstrates restored neurological behavior, diminished lesion zones, and an attenuated inflammatory response following IL-1 modulation either through IL-1 knock-out experiments, IL-1β inhibition, or IL-1ra treatment. Of these, IL-1ra treatment is likely the most physiological. In addition, recombinant human IL-1ra (anakinra) is already approved for utilization across a few rheumatologic disorders. As of today, one randomized clinical controlled trial has utilized IL-1ra inhibition as an intervention and demonstrated its safety. Further clinical trials powered for patient outcome are needed in order to demonstrate efficacy. In this review, we summarize IL-1 biology in relation to acute neuroinflammatory processes following TBI with a particular focus on current evidence for IL-1ra treatment both in the experimental and clinical context.

## Introduction

Traumatic brain injury (TBI), i.e., altered cerebral function due to external physical force [1], is a heterogenous disorder encompassing mild, moderate, and severe injuries [2]. TBI is a globally important cause of mortality or life-long disability [3] and currently, 55 million people are estimated to live in the aftermath of TBI [4]. Following decades of sparse interest, TBI is emerging as a pivotal health priority, not least because of the putative association between neurodegenerative diseases and TBI [5, 6]. International collaborative efforts, including the Collaborative European NeuroTrauma Effectiveness Research study (CENTER-TBI) [7] and the Transforming Research and Clinical Knowledge in TBI (TRACK-TBI) [8, 9], have used large aggregated observational datasets to identify promising strategies for patient stratification using a range of biomarkers, clinical, radiological, and biochemical. The hope is that this will allow patient-tailored treatment (so-called personalized medicine), by accurately identifying the specific pathological abnormalities that occur in a given patient [3]. With this in mind, it is likely that the next phase of TBI research will be pathophysiology-oriented studies that target these specific processes.

Following the injury eliciting trauma (also known as the primary injury), TBI evokes numerous cellular and humoral injury processes. If left untreated, these may cause an irreversible secondary brain injury [10, 11]. Conversely, these mechanisms also constitute therapeutically tractable targets. The plethora of cellular injury processes include disruption of ion homeostasis, excitotoxicity, edema, blood–brain barrier (BBB) disruption, and inflammation [10]. Among these, the inflammatory responses elicited in the injured brain have gained particular interest [12]. Whereas the central nervous system (CNS) historically was believed to be immune privileged, current experimental data support a distinct *neuroinflammatory* response elicited by the trauma [13]. Some of these reactions are likely to be detrimental, while others are beneficial to the injured brain [14, 15]. As such, an understanding of the inflammatory response to TBI has become a specific focus for both pre-clinical and clinical researchers [12].

Neuroinflammation is initiated in the immediate vicinity following the trauma, as a consequence of both local cell death, vascular injury, and BBB disruption [13, 16]. Initially, innate immune mechanisms are activated [15]. This triggers a cascade of events that leads to successive recruitment of various immune-related mediators until adaptive responses ensue weeks to months following the initiating insult [13, 14]. Cytokines, small (~ 20 kDa) proteins that serve as inflammatory mediators synthesized across immune but also CNS cells [17, 18], are critical regulatory mediators in these processes [16], as shown across vast numbers of experimental studies [17]. In human severe TBI, cytokine increments have been described across CNS compartments of both brain extracellular fluid and cerebrospinal fluid (CSF). In this context, the interleukin- (IL-) 1 family is among the most studied cytokines [17, 19]. This cytokine family entails two agonistic ligands, namely IL-1α and IL-1β as well as an antagonist, i.e., the IL-1 receptor antagonist (IL-1ra) [20]. Following experimental discoveries that IL-1 inhibition following TBI is beneficial, IL-1ra-mediated IL-1 inhibition has been hypothesized to be a feasible avenue for neuroinflammatory modulation following TBI [21]. Below, we summarize IL-1 biology and signaling in the CNS, followed by a detailed portrayal of IL-1 contextualized to the acute neuroinflammatory events that ensue TBI. In addition, we review the current state of the experimental and clinical research on IL-1ra treatment following TBI.

## Interleukin-1 Is a Core Mediator of Innate Neuroinflammation Amenable to Pharmacologic Modulation

IL-1 was discovered in the 1940s as the body’s endogenous fever-causing mediator [22, 23]. In 1974, it was denominated *lymphocyte-activating factor* [24], a nomenclature which was swapped to the IL-system in 1979 [25]. Since then, the knowledge of IL-1 has broadened into a big protein family of strictly regulated mediators. Current IL-1 agonists entail IL-1 (referring to both IL-1α and IL-1β [22, 26]), as well as IL-33, IL-36α, IL-36β, and IL-36γ [27]. These agonists bind the type I IL-1 receptor (IL-1R1), which mediates the majority of IL-1 induced signaling-related effects [22, 28, 29]. There is also a type II IL-1 receptor (IL-1R2), which however constitutes a so-called decoy receptor [27], which means that it inhibits IL-1β signaling [22] by binding the protein while lacking the domains for intracellular transduction. In addition, the IL-1 family is also composed of proteins with antagonistic effects to IL-1 and IL-1R1 [22, 27]. The best known among the antagonists, IL-1ra, functions as an endogenous competitive inhibitor [22] through binding to IL-1R1 and thereby hindering activation of the receptor by IL-1α or IL-1β [30].

**Fig. 1:**
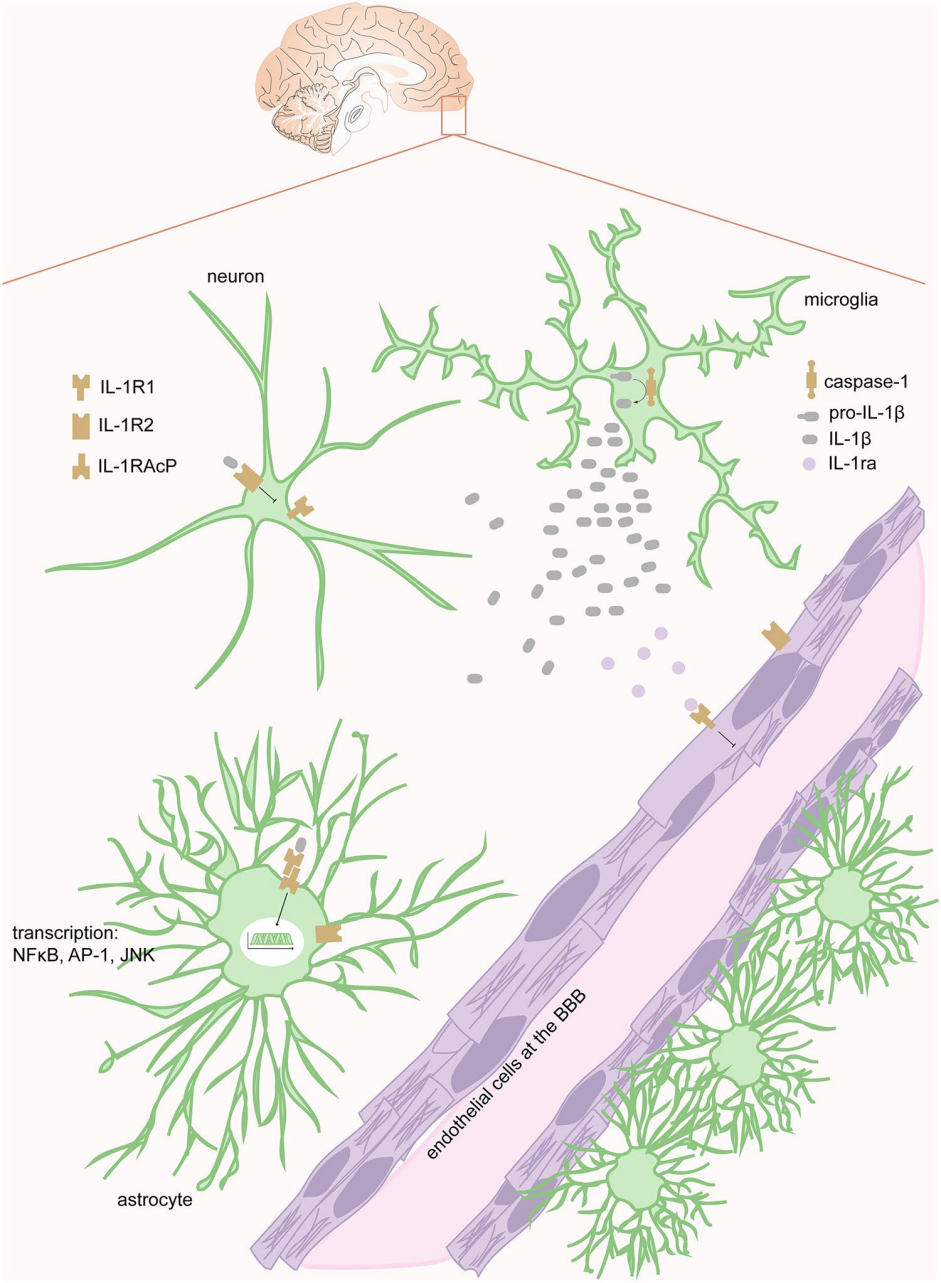
**IL-1 signaling in the CNS.** IL-1 ligands such as IL-1α, IL-1β, and IL-1ra binds to either one of the two IL-1 receptors IL-1R1 or IL-1R2. IL-1β is synthesized upon inflammasome activation, typically within microglia and released. Upon binding to IL-1R1 (located at neurons, astrocytes, and endothelial cells), IL-1β elicits an intracellular signaling cascade, dependent on dimerization to the IL-1 receptor accessory protein. Ultimately, this leads to transcription of mediators pertaining to various pathways, e.g. NFκB, AP-1, and JNK. IL-1ra is a competitive IL-1 antagonist and hinders further IL-1 signaling. IL-1R2 is a decoy receptor, which also inhibits IL-1 signaling. **Abbreviations:** AP-1, activator protein 1; IL-, interleukin; IL-1R, interleukin receptor type; IL-1ra, IL-1 receptor antagonist, IL-1RAcP, IL-1 receptor accessory protein; JNK, c-Jun N-terminal kinase; NFκB, nuclear factor kappa light-chain-enhancer of activated B cells

Under homeostatic conditions, IL-1 ligands are expressed at low levels [26] but IL-1 transcription is rapidly induced following a broad range of stimuli [26]. To become active, IL-1β requires caspase-1-mediated cleavage while IL-1α does not [31]. The key regulator in this process is the inflammasome [23], highlighted in the TBI context in detail below. Upon binding, another IL-1 receptor accessory protein causes receptor dimerization, whereby the intracellular signaling cascades ensue. This ultimately leads to protein recruitment that, coupled with protein kinases, activates transcription factors pertaining to the nuclear factor kappa light-chain-enhancer of activated B cells (NFκB), activator protein-1, c-Jun N-terminal kinase, mitogen-associated protein kinases, and p38 pathways among else [22, 31]. The joint effect of these signaling events is an all-encompassing activation of innate immunity [23]. These events are schematically summarized in Fig. 1.

### IL-1β Is Expressed Across the CNS, Exerts Important Homeostatic Functions, and Might Be Attenuated Following Insult

All subsets of recognized IL-1 mediators have expression within the CNS (Fig. 1) [26]. Microglia, however, constitutes the key source of IL-1 production in the CNS before leukocyte infiltration [23], where pro-IL-1β is mostly located within the cytoplasm [31]. Under homeostatic conditions, the IL-1 family is likely to be involved in normal CNS functions, e.g., neuronal signaling, ionic homeostasis, synaptic plasticity, long-term potentiation, sleep regulation, induction of neurotrophic factors, and adult neurogenesis [23, 26, 31]. This diverse set of functions is likely enabled through cell type specific IL-1R1 signaling pathways within the CNS [29]. Recent data indicate that endothelial cells, astrocytes, neurons, choroid plexus cells, and ependymal cells express IL-1R1 [28, 29]. Notably, microglia do not seem to express IL-1R1 [29]. Instead, in recent work [29], microglial activation was claimed to be indirectly mediated through IL-1-mediated activation of endothelial cells, as well as ependymal/choroid plexus cells [29]. These cells also exerted other effector consequences downstream of IL-1 stimulation such as leukocyte/monocyte recruitment and pro-inflammatory cytokine release [29].

Given the versatile involvement of IL-1 in various inflammatory cascades and pathways, IL-1 has been postulated as a therapeutically controllable master-regulator of inflammation. Since the 1990s, it has been observed that attenuated IL-1 expression in the CNS following traumatic and non-traumatic neuroinflammatory conditions seems beneficial [26]. Theoretically, IL-1 inhibition can be obtained through inhibition of the maturation/cleavage of pro-IL-1β, inhibition of extracellular IL-1β, and inhibition/antagonism of the IL-1R1 [22]. Utilization of the endogenous competitive antagonist IL-1ra is the most widely studied [26]. In vivo, IL-1ra binds to IL-1R1 without protein dimerization, thus inhibiting downstream activities [27]. IL-1ra has been developed into a pharmaceutical substance—human recombinant IL-1ra (rhIL-1ra, anakinra). Anakinra has been tested across several rheumatologic disorders [22, 32]. Today, the substance is approved for use in patients with rheumatoid arthritis or cryopyrin-associated periodic syndrome [26, 27]. In CNS disorders, rhIL-1ra has been utilized in randomized studies of both aneurysmal subarachnoid hemorrhage and stroke [33–35]. In this work, rhIL-1ra has been shown to diminish inflammatory responses, and be safe [34, 35]. One study, which failed to recruit in accordance with their power analysis [33], could not demonstrate a reduction in neuroinflammation following rhIL-1ra treatment.

Taken together, the IL-1 family constitutes a collection of upstream innate immune mediators of core importance for neuroinflammation. Likely, the IL-1 family’s intimate regulation with microglia is indirect through versatile signaling mechanisms across several CNS cell types. Therapeutics utilizing knowledge of the IL-1 system has already been implemented in rheumatology, but data is beginning to emerge for other CNS disorders. Following studies in non-traumatic acute brain injury, initial data has shown reduction in inflammation and safety. The remaining discussion is focused on TBI, both from the experimental and clinical viewpoint.

## Innate Neuroinflammation Following Traumatic Brain Injury

**Fig. 2:**
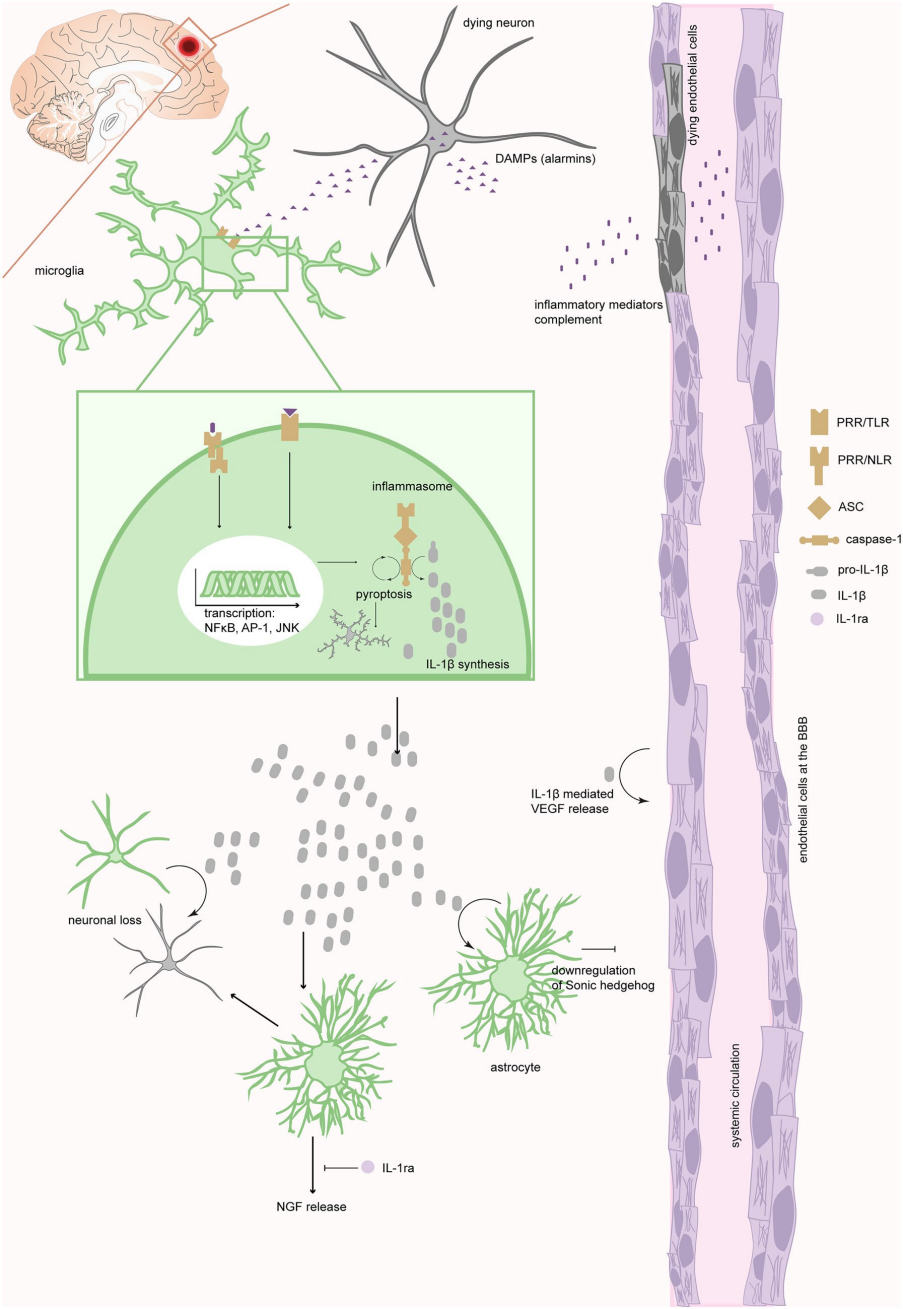
**IL-1 synthesis and downstream effects following traumatic brain injury.** Upon trauma, tissue destruction leads to the release of DAMPs from dying cells and leakage of inflammatory mediators such as complement across a disrupted BBB. This elicits innate CNS immune responses through binding of DAMPs to PRRs. Across these events, microglia is a core CNS specific immune cell. Various PRRs elicit different immune responses. In the inset, a priming and activation stimulus are depicted, typically necessary to activate the inflammasome within microglia. This yields cleavage of pro-IL1β into its active form. Inflammasome activation can also yield pyroptosis, through which even more IL-1β is expected to leak into the extracellular room. Here, IL-1β effectuates numerous biological processes, of both deleterious and beneficial character for the injured CNS. **Abbreviations:** AP-1, activator protein 1; ASC, apoptosis-associated speck-like protein containing a caspase recruiting domain; BBB, blood-brain barrier; CNS, central nervous system; DAMP, damage-associated molecular pattern; IL- interleukin; IL-1ra, IL-1 receptor antagonist; JNK, c-Jun N-terminal kinase; NGF, nerve growth factor; NFκB, nuclear factor kappa light-chain-enhancer of activated B cells; NLR, nucleotide-binding oligomerization domain-like receptor; PRR, pattern recognition receptor; TBI, traumatic brain injury; TLR, Toll-like receptor

Below, we provide a general overview of the chronological sequence of neuroinflammatory events that ensue following TBI, focusing first on core receptors and secondly on CNS inflammatory cells. We specifically highlight pathways where IL-1β is a core inflammatory mediator following TBI. Basic IL-1 biology is described above, while this section contextualizes IL-1β specifically in TBI. The discussion is centered at the acute phase following severe TBI. The sequence of events described below are schematically summarized in Fig. 2.

### The Initiating Trauma Triggers Release of Damage-Associated Molecular Patterns that Bind to Cell-Specific Pattern Recognition Receptors

Immediately upon trauma, cell death and injury ensue. At the CNS borders, such as the BBB, loss of tissue integrity leads to leakage from the periphery of among else inflammatory mediators and complement [14, 16, 36]. Within the parenchyma [14, 16, 36], local tissue injury leads to the emergence of damage-associated molecular patterns (DAMPs) [13], entailing otherwise intracellular molecules that reach the extracellular milieu [37], e.g., as a consequence of tissue necrosis and cytoplasmic leakage interstitially. Following (sterile) trauma, the subgroup of DAMPs that are organism-endogenous are referred to as alarmins [38]. These include but are not limited to high mobility group box1 (HMGB1), heat shock proteins, S100 proteins, IL-1α, IL-33, uric acid, and adenosine triphosphate.

Alarmins function as ligands to pattern recognition receptors (PRRs), a collective name for several receptor families with different stereotypical downstream signaling pathways, as well as cellular localizations [13]. Specific subfamilies of PRRs include Toll-like receptors (TLRs), nucleotide-binding oligomerization domain-like receptors (NLRs), RIG-like receptors, absent in melanoma-2-like receptors, and other receptor families [39]. Specific subclasses of PRRs utilize similar downstream signaling pathways [13]. In line with this, the most studied receptor—TLRs—can be expressed both at the cell surface or the cytoplasm [40, 41]. These receptors most commonly signal through either myeloid differentiation factor 88 which leads to NFκB activation or (in case of TLR3) the TIR domain-containing adaptor protein-inducing interferon β [39, 41, 42]. In the CNS, all cell types likely express some subtypes of TLRs, whereas microglia express all known TLR subtypes [40, 43]. This seems natural as microglia serve as the surveillance cell in the CNS and is the first cell-type to become activated [12, 44], through among else DAMP-mediated activation of TLRs, especially TLR4 [42]. Importantly, astrocytes also express TLRs and promote, e.g., cytokine production, astrocytic migration, and reduce neuronal survival [41].

In contrast to TLRs, NLRs are exclusively expressed intracellularly [40], and signaling results in assembly of the inflammasome [12], crucial for caspase-mediated cleavage of pro-IL-1β into its active form [45]. Because of its central role for IL-1β, we next discuss the inflammasome specifically.

### Inflammasome Assembly and Activation Following TBI Is Crucial for IL-1β Production, While Inflammasome Overactivation Can Lead to Pyroptosis

The inflammasome, originally described in 2002, is a critical part of the innate immune response [45]. Generally, inflammasomes can be defined as large multiprotein complexes (estimated to ~ 700 kDa) consisting of three unique units: a PRR, an adaptor protein called apoptosis-associated speck-like protein containing a caspase recruiting domain (ASC), and caspase 1 [13, 40, 46]. The PRR can consist of, e.g., an NLR or Absent in melanoma 2-protein [6, 40]. Currently, at least six different inflammasomes utilizing NLRs as sensor proteins have been described [40]. The first inflammasome subtype described—NLR family pyrin domain containing (NLRP) 1 [46]—is expressed in cerebral cortex neurons and microglia [14, 47, 48]. In addition, NLRP2 and NLRP3 have been described in the CNS [40]. Astrocytes have been shown to express NLRP2 [49], while the NLRP3 inflammasome has been implicated in astrocytes, microglia, and neurons [44, 50].

For the inflammasome to become activated, a priming stimulus followed by an activating ditto is warranted [51]. The priming stimulus and downstream activating signaling mechanism is unique to the inflammasome subtype [6]. For example, NLRP3 can be primed by several stimulus which converge into NFκB signaling, after which activation ensues through yet incompletely described mechanisms [52]. Common to the various inflammasome subtypes are the downstream effector events, which can be subdivided into pyroptosis or IL-1β and IL-18 release [45, 46]. Pyroptosis has been associated with inflammasome overactivation [51] and is a distinct cell death mechanism, whereby cell lysis occurs and is followed by release of cellular content otherwise restricted to the cytoplasm [46], thus plausibly further incrementing inflammatory signaling through release of pro-inflammatory cytokines [44]. Notably, this can be one mechanism through which IL-1β and IL-18 are released from the cytoplasm [52]. Pyroptosis has been described in neurons and glial cells [44]. Conversely to pyroptosis, inflammasome activation also leads to caspase-1-mediated cleavage of pro-IL-1β into IL-1β, and analogously for IL-18 [40]. This is then followed by extracellular release, which is still incompletely described [46]. In accordance with this, NLRP3 activation is seen already at 6 h following TBI [50] and IL-1β has been seen to increase also as early as 15 min [48], 4 h [53], and 6 h [50] following trauma. In summary, inflammasome-induced caspase-1-mediated cleavage is a core mechanism through which IL-1β is synthesized in the CNS following TBI. Incremented and abnormal inflammatory stimulation leads to pyroptosis, potentially leading to even higher levels of IL-1β interstitially.

### Downstream Effects of IL-1 Signaling Entail Presumed Propagation of Inflammatory Signaling, Encompassing Both Neurotoxic and Protective Mechanisms

IL-1β is thus rapidly induced following trauma onset and inflammasome activation. In fact, the entire IL-1 family of cytokines are among the earliest innate immune mediators to emerge [13, 26]. After exerting the intracellular cascades as described above, IL-1β propagates the post-traumatic pro-inflammatory response in multi-faceted ways. For example, IL-1β contributes to BBB disruption following TBI [54, 55]. It has been suggested that this mechanism is mediated through IL-1-signaling-induced release of vascular endothelial growth factor [56], but also through IL-1β-mediated downregulation of astrocytic Sonic hedgehog [57], or IL-1β-mediated secretion of other cytokines, which in turn act on the BBB [55]. In line with this, the ratio between IL-1β expression in CSF and blood has been correlated with BBB integrity in patients following severe TBI [58].

IL-1β has also been shown to diminish signaling of brain derived neurotrophic factor (BDNF) [59], a core neurotrophin for CNS neurons [60]. This is of importance in the TBI context, where polymorphism in the BDNF gene has been associated with cognitive capacity following trauma [61]. Moreover, IL-1β has also been suggested to mediate neuronal loss after TBI [54]. In vivo, following experimental TBI, this has been suggested to occur through extracellular signal-regulated kinase-dependent phosphorylation [62]. More recently, astrocytes were suggested to exert neurotoxic effects following astrocytic stimulation of IL-1α rather than IL-1β [63]. The mechanism underlying this finding was later suggested to be saturated lipoparticles [64]. In contrast, we recently derived brainstem astrocytes from embryonic stem cells and subjected them to similar cytokines in vitro as described in Ref. [63]. We could induce the neurotoxic effect on motor neurons through utilization of these cytokines, but also through utilization of IL-β and IL-6 jointly [65]. This mechanism, shown to be mediated through pathways related to endoplasmic reticulum stress and altered regulation of MYC [65], could possibly be of interest also in the in vivo context after TBI. In addition to these highlighted downstream functions of IL-1β, this pro-inflammatory cytokine is likely also involved in peripheral immune cell recruitment, edema formation, initiation of phagocytosis, and cytokine production to name a few [54, 66]. Taken together, IL-1 downstream signaling elicits broad downstream consequences that propagates deleterious aspects of inflammation following TBI.

In contrast, IL-1β signaling also seems to be involved in presumptive neuroprotective responses elicited after TBI. Both intracerebroventricular injection of IL-1β as well as experimental TBI models increase the expression of nerve growth factor in the CNS, suggested to be released from astrocytes [67, 68]. In line with this, treatment with IL-1ra reduces nerve growth factor release following TBI [69], thus suggesting that IL-1β also likely confers neuroprotective effects. In summary, the duality of IL-1β suggests that even if it is a tentative treatment target [26] following TBI, the multi-faceted effects downstream of IL-1β might yield unexpected and undesirable hindering of neuroprotective mechanisms.

### Cellular Immune Events Occurring Across the CNS in Parallel to IL-1β Cleavage Promotes Peripheral Immune Cell Recruitment and Adaptive Immune Responses

The discussion above is centered around IL-1β, while naturally there are numerous other pro-inflammatory events happening in conjunction to inflammasome activation. Microglial activation is synchronously promoted by other inflammatory mediators, such as complement [36, 70]. Complement serves as a molecular target of interest for modulation of the inflammatory response after experimental TBI [70], and a clinical trial of complement inhibition is ongoing [71]. Activated microglia also exert a plethora of functions, including cytokine production [16, 72] of, e.g., IL-6, tumor necrosis factor-(TNF-)α, interferon-γ, and reactive oxygen species [12, 16]. Astrocytes act in concert with microglia, as described recently in response to microglia-mediated cytokine production [63]. Microglial activation also leads to the production of chemokines [72], i.e., small, heparin-binding proteins which serve as leukocyte attractants [17]. Activated astrocytes further promote the release of chemoattractants [13]. This leads to the recruitment of peripheral immune cells to the site of injury [72]. In fact, the first peripheral immune cells arrive within hours to the lesioned brain and consist of neutrophils [12, 13]. Throughout the ensuing days, they are accompanied by monocyte-derived macrophages, marking late innate immune reactions [13, 14]. After several days stretching into weeks, adaptive immune responses begin to emerge [13].

## Interleukin-1 Receptor Antagonist Treatment Shifts the Inflammatory Response Intracranially

A substantive body of evidence has demonstrated IL-1β increments following TBI in both the experimental and clinical context [17]. It has for some time been hypothesized that IL-1ra confers a neuroprotective effect in the aftermath of TBI [73]. Below, we summarize experimental and clinical work on IL-1 modulation with a particular focus on IL-1ra-based therapeutics. Readers are also referred to another up-to-date review on these matters [74].

### IL-1 Modulation Following Experimental TBI Reduces Lesion Size, Attenuates Pro-inflammatory Signaling, and Improves Functional Outcome

Experimental work exploring IL-1-modulation following experimental TBI has been undertaken for decades. In Table 1, selected works are summarized. Works included in this table all demonstrate specific IL-1β modulation. We have also included work pertaining to specific upstream inflammasome/inflammasome subset inhibition [48, 75–77], while excluding studies utilizing non-specific mediators, of which one effect might be inflammasome inhibition [78–80]. Moreover, we have included genetic knock-out studies on the IL-1R1 [81–83], as well as IL-1α and IL-1β inhibition [82]. Naturally, we also include studies utilizing pharmacologic modulation of IL-1β, either through utilization of IL-1ra [82, 84–88] or through neutralizing antibodies [89–94]. Taken together, *n* = 20 studies were found. One employed an aseptic cryogenic injury model [95] instead of the more traditional mechanical injury models, of which the most common was the fluid percussion injury model [96, 97]. Among all included studies, *n* = 6 were carried out in rats, while *n* = 14 were carried out in mice. Unexpectedly, in *n* = 6 studies [69, 76, 81, 92, 93, 95], the authors did not specify injury severity. This seems rather striking from a translational viewpoint, where patient disease trajectories are expected to be highly dependent on trauma endotypes [98], but also the experimental context, where different trauma types yield different inflammatory responses [99].

Principally, four methods for IL-1 modulation were employed across the different studies, in accordance with the theoretical line of reasoning stated above [22]. First, IL-1 inhibition can be achieved through inflammasome modulation upstream of the IL-1R1. Two [76, 77] of the studies [48, 75–77] directed at inflammasome inhibition incorporated behavioral testing. Here, mice improved both some motor and cognitive skills following inflammasome inhibition [76, 77]. In parallel, these studies demonstrated diminished lesion volumes [48, 76, 77] and attenuated cerebral edema [76, 77]. All of these studies also modulated the inflammatory response, including IL-1β [48, 75–77]. These findings are in line with studies utilizing non-specific immune modulation following TBI, where one of presumably several effects is inflammasome/IL-1β attenuation [78–80]. Of particular interest among non-specific inflammasome modulators is hypothermia. Early work [100] demonstrated that hypothermia decreased IL-1β levels following TBI and at the same time normalized nerve growth factor expression. Later work [101] observed among else inflammasome modulation following hypothermia, while others have argued that hypothermia exerts its effect(s) through TLR-4-mediated myeloid differentiation factor 88 signaling [102]. Hence, the exact mechanisms mediated through hypothermia are still incompletely characterized. This is reflected in a clinical trial on hypothermia following TBI [103], where hypothermia was only seen to be beneficial in limited subgroup analyses. In line with this, hypothermia is only advised as a last-tier therapy following clinical TBI [104]. One possible mechanism for this might be that hypothermia inhibits neuroprotective mechanisms in adjunct to its anti-inflammatory effects, in a fashion similar to how nerve growth factor is inhibited following treatment with IL-1ra [69].

Next, IL-1 modulation has also been studied through genetic modulation [81–83]. Here, knockout of the IL-1R1 alters the CNS endogenous and peripheral immune response following injury [81], without necessarily alleviating cerebral edema or lesion volumes [82, 83]. Interestingly, despite the lack of structural findings, neurological outcome of the animals improved in the majority of tests following IL-1R1 deletion in one of the studies undertaken [83].

Lastly, IL-1 modulation can also be obtained through inhibition of the IL-1R1 utilizing either IL-1β antibodies [89–94] or else IL-1ra in either the human or mouse recombinant form [82, 84, 85, 87, 88]. In addition, two studies [69, 86] utilized genetically induced IL-1ra overexpression in situ. With the exception of genetical IL-1ra overexpression, the route of administration of the IL-1 modulator must be considered. Among the included studies, the IL-1 modulator was administered subcutaneously [85, 87, 88], intravenously [85], intraperitoneally [90–93], and intracerebroventricularly [84, 89, 94, 95]. This is particularly important when considering IL-1ra, given the poor BBB penetrance of this molecule [26, 74].

In some studies, IL-1ra or IL-1β antibodies ameliorated behavioral changes following TBI including motor functions [95], whereas other studies rather found cognitive improvements and complex behavioral changes [85, 89–91]. Among the included studies, one specifically assessed post-traumatic epilepsy [87] and found acutely and chronically diminished seizure susceptibility. The causal mechanism underlying these behavioral findings likely pertain to diminished lesion magnitude. In line with this, several studies report an attenuated inflammatory response following IL-1ra or IL-1 antibody treatment [87–89, 92, 93, 95]. Even though this per se is not necessarily prognostically beneficial, the same studies show simultaneous lesion volume diminishment, fewer dying neurons, diminished edema, attenuated caspase 3-expression, reduced oligodendrocyte loss, preserved parvalbumin interneurons, and dopaminergic signaling [84, 85, 87–90, 92–95]. Taken together, ample experimental evidence suggests a role for IL-1 modulation following TBI.

**Table 1:** Experimental IL-1 modulation following traumatic brain injury

**Ref.**	**Year**	**Animal**	**TBI model**	**Injury severity**	**Intervention**	**IL-1β specific **	**Administration route**	Structural findings*	Functional results*	Inflammatory results*	**Ref. number**
Toulmond et al	1995	rats	lateral fluid percussion injury	moderate	rhIL-1ra	yes	Intracerebro-ventricularly	attenuated lesion size	NA	NA	[84]
Dekosky et al	1996	rats	-cortical stab wound injury -weight-drop	NA	*in situ* overexpression human IL-1ra	yes	transplantation of genetically modified rabbit fibroblasts	qualitatively reduced microglial infiltration	NA	- weight drop: reduced nerve growth factor levels compared with lesioned non-injected animals. - stab wound: lower nerve growth factor expression compared with vehicle, vector only but not with unlesioned animals.	[69]
Sanderson et al	1999	rats	lateral fluid percussion injury	moderate	rhIL-1ra	yes	intravenous and subcutaneous	attenuated neuronal loss	-high dose rhIL-1ra: worse motor function at 7 days post injury, but better cognitive outcome	NA	[85]
Basu et al	2002	mice	penetrating brain injury	NA	Genetic knockout: IL-1R1	yes	-	-decreased number of endogenous microglia, CNS recruited macrophages -decreased cyclooxygenase-2, decreased Vascular cell adhesion molecule 1 -decreased astrogliosis	NA	-reduced levels of:IL-6 -unchanged: TNF-α	[81]
Tehranian et al	2002	mice	closed head injury model	mild^a^	transgenic overexpression of IL-1ra in astrocytes	yes	-	diminished edema	-less post-traumatic impairment with regard to motor tasks and neurological severity scores (inconclusive)	-increased mRNA levels: IL-1β -decreased protein levels: IL-1β -increased levels: TNF-α -modified levels: IL-6	[86]
Jones et al	2005	mice	aseptic cryogenic injry	NA	rmIL-1ra	yes	Intracerebro-ventricularly	-diminished lesion volume -fewer dying neurons	Improved results beam balance score, grid test	reduced number of nitric oxide synthase-expressing cells	[95]
Lu et al	2005	rats	weight drop	severe†	Antibody to: IL-1β/IL-1α	yes	Intracerebro-ventricularly	attenuated neuronal loss	NA	NA	[94]
Clausen et al	2009	mice	controlled cortical impact	mild-moderate	IL-1β antibody	yes	Intracerebro-ventricularly	-diminished lesion size	-no difference rotarod, no difference cylinder test -improvement Morris-Water-Maze	-diminished microglia response -reduced neutrophil recruitment -reduced T-cell infiltration -	[89]
De Rivero Vaccari et al	2009	rats	fluid percussion injury	moderate	antibodies against ASC	no	Intracerebro-ventricularly	-Decreased brain contusion volume.	NA	-attenuated inflammasome protein expression (including caspase-1, IL-1β).	[48]
Clausen et al	2011	mice	controlled cortical impact	mild-moderate†	IL1β antibody	yes	intraperitoneally	-Lower brain water content/diminished edema. -No difference in the number of activated microglia. -Decreased tissue loss.	No difference in memory retention, improved long-term visuospatial learning, no difference in the memory probe trial, no difference rotarod, no differences cylinder test	No difference in Gfap, Il-6, Ccr2, Ccl3	[90]
Ekmark-Lewén	2016	mice	central fluid percussion injury	mild-moderate^a^	IL-1β antibody	yes	intraperitoneally	No differences in the number of microglia/macrophages or ventricular size	Improved results in the multivariate concentric square field test, Morris-Water-Maze	NA	[91]
Semple et al	2017	mice^b^	controlled cortical impact	moderate-severe	rhIL-1ra	yes	subcutaneously	-smaller cortex volume loss,	lower seizure severity scores, diminished long-term cognitive deficits, protective effect on chronic seizure susceptibility	- attenuated long-term astrogliosis - decreased hippocampal GFAP immunofluorescence - decreased Gfap and Vimentin gene expression	[87]
Sun et al	2017	mice	closed head injury model^c^	mild	rhIL-1ra	yes	subcutaneously	increased brain edema, -reduced post-traumatic brain atrophy, -increased white matter integrity	No differences	-Attenuated macrophage/microglial/cytokine activation - decreased astrocytic response - reduced leukocyte infiltration	[88]
Flygt et al	2018	mice	central fluid percussion injury	NA	IL-1β antibody	yes	intraperitoneally	-Reduced caspase-3 expression, -attenuated loss of oligodendrocytes	-	-attenuated microglial/macrophage activation, -altered microglia morphology	[92]
Newell et al	2018	mice	lateral fluid percussion injury	moderate, severe	Genetic knockout of: IL-1α, IL-1β, IL-1R1 or rh IL-1ra	yes	intraperitoneally	IL-1 genetic variations: no differences in lesion volumes	-Genetic: no differences on rotarod testing; IL-1R1-/- performed better in the Barnes Maze test. -rhIL-1ra resulted in improved learning at 3 days following injury	-IL-1β-/- expressed lower IL-1α levels -IL-1R1-/- leads to multiple cytokine level alterations, changes duration of inflammatory increments. -rhIL-1ra decreased IL-1β levels.	[82]
Xu et al	2018	mice	controlled cortical impact	moderate	MCC950 (NLRP3 inhibitor)	no	intraperitoneally	-Reduced brain edema. -Diminished lesion volume. -Reduced apoptosis	-Modified Neurological Severity Score, Rota-rod improved. -Some aspects of Morris-Water-Maze improved.	-Altered composition of immune cell recruitment, altered soluble cytokines - Decreased levels inflammasome components (including caspase-1 and IL-1β).	[77]
Chung et al	2019	mice	controlled cortical impact or closed head injury model	mild-moderate^a^	IL-1R1 deletion	yes	-	-Closed head injury model: does not induce edema, blood-brain barrier disruption. -CCI: No difference between edema, lesion volume between wild-type and IL-1R1 knock-out.	-Closed head injury model: IL-1R1 knock-outs had better performance Morris-Water-Maze, Y maze. -CCI: improved wire-grip performance, worse performance Morris-Water-Maze, no difference Y maze or proble trials	CCI: similar increase in CD11b+/CD45+ leukocytes, brain lymphocytes.	[83]
Kuwar et al	2019	rats	controlled cortical impact	moderate	JC124 (NLRP3 inhibitor)	no	intraperitoneally	-Attenuated cortical damage. -Improved neuronal survival. -Decreased caspase-1 levels (pro-caspase-1, activated caspase-1)		-NLRP3 and ASC decrease -Reduced IL-1β in serum and focally; -Reduces other inflammatory mediators	[75]
Ozen et al	2020	mice	central fluid percussion injury	authors do not specify	IL-1β antibody	yes	intraperitoneally	-Preserved parvalbumin+ interneurons in the basal ganglia, -normalized dopaminergic innervation.	-	-Diminished microglial activation	[93]
Yan et al	2020	mice	closed head injury model	authors do no specify	Oridonin (covalent NLRP3 inhibitor)	no	intraperitoneally	-Alleviation of cerebral edema and diminished BBB disruption. -Attenuated tissue loss -Improved neuronal survival/reduced apoptosis.	Improved performance post-TBI on Modified Neurological Severity Score, Rota-rod, Hanging wire test	Inflammasome subsets and caspase-1 inhibited by Oridonin, IL-1β and IL-18 diminished.	[76]

Experimental data depicting IL-1β modulation following experimental, preclinical TBI since the mid-2000s. Both lesion size, inflammatory quantifications and behavioral findings seem to be improved following IL-1 inhibition

* compared with vehicle/wild-type

a As per original definitions described in Refs. [97, 105–107]

b pediatric TBI

c polytrauma

### Clinical Studies of IL-1 Following TBI

The activation and incremented levels of the IL-1 family members including IL-1 and IL-1ra are well-documented in the CNS following TBI, as reviewed in Ref. [17]. Different protein quantification techniques hold promise for protein biomarker discovery in CSF following trauma [108]. In a uniquely large cohort of patients, Lindblad and colleagues assessed *n* = 177 proteins observationally following human severe TBI across both CSF and blood. As expected, both IL-1α and IL-1β demonstrated an increased expression in CSF [58]. Moreover, these proteins were also shown to be significantly associated with BBB disruption, thus pointing towards an important interplay between these two cellular injury mechanisms following TBI [58, 109]. In addition to CSF, cytokine production has also been assessed utilizing cerebral microdialysis. Following TBI, cytokine production likely exhibits a stereotyped sequential expression temporally. Throughout this process, expression levels of IL-1α, IL-1β, and IL-1ra are believed to co-vary [110]. Importantly, substantive data suggest that the production of these cytokines occur also within the human CNS [111], but with influences from the periphery. The latter has been demonstrated in TBI patients with non-CNS infections, where the peripheral immune response shifts CNS production of inflammatory mediators [112]. Notably, brain extracellular fluid levels of IL-1ra decreased in this patient group [112]. In blood, numerous structural biomarkers are intensively studied, among else glial fibrillary acidic protein, S100B, neurofilament light, ubiquitin C-terminal hydrolase L1, tau, and neuron-specific enolase. In blood, these biomarkers have been shown to serve as surrogate markers of brain injury burden [113], but not more distinct anatomical pathology [114]. In line with this, inflammatory modulation specifically directed at microglia results in altered neurofilament light values [115], possibly indicating that specific blood biomarkers might serve utile in the future.

Following these observational studies, the next step is to delineate whether the inflammatory response, and specifically IL-1 and IL-1ra, affects clinical outcome. In several studies, outcome analysis has been precluded due to small sample size [21, 112], hence why data is sparse in this domain. A recent systematic review investigated protein biomarkers in CSF following TBI and found several proteins associated with outcome. No studies assessing IL-1ra or other IL-1 proteins were included in this review, and IL-1ra did therefore not show either a beneficial or deleterious effect on outcome [116]. In contrast, Zeiler et al. reviewed cytokines in both CSF and cerebral microdialysis following human TBI [19]. Here, the authors found *n* = 4 studies [117–120] demonstrating a relationship between IL-1β in CSF and patient functional outcome. The discrepancy between the results of these two systematic reviews is likely a consequence of broader inclusion criteria as Zeiler and colleagues included pediatric studies [117, 118], one study that demonstrated a borderline significant trend between CSF-IL-1β and outcome [119], as well as one study predominantly describing increased CSF levels of IL-1β without a clear relationship to outcome [120]. In contrast, among cerebral microdialysis studies, there has been but one study [73] which has demonstrated a relationship between IL-1ra and functional outcome.

To date, one phase II randomized controlled trial assessing recombinant human IL-1ra has been undertaken [21], the primary outcome of which was safety assessment, while demonstrating feasibility and an altered neuroinflammatory response. rhIL-1ra was administered subcutaneously in doses of 100 mg once daily throughout 5 days from injury. First, rhIL-1ra was a safe study drug as per a priori definitions throughout the study protocol. Importantly, the authors demonstrated that the study drug reached the CNS and maintained an adequate concentration within the CNS throughout the study period. Utilizing principal component analysis, the authors also demonstrated a neuroinflammatory shift, further speaking in favor of the treatment effect.

In a follow-up study [121] utilizing the same clinical cohort, the neuroinflammatory response was characterized in greater detail through advanced statistical tools. Uniquely, the authors demonstrated a CNS-specific, temporally regulated shift in cytokine expression. Cytokine responses were interpreted in accordance with the then dominating paradigm for microglial responses [122, 123], which described microglia as polarized towards either a pro- or anti-inflammatory state. Today, this concept is largely abandoned as microglial response has been shown to be more versatile [124]. In this study by Helmy et al. [121], rhIL-1ra treatment was found to shift the neuroinflammatory response in both brain extracellular fluid and in plasma. For brain extracellular fluid, the neuroinflammatory shift predominantly occurred within the first 48 h following injury. Intracranially, the rhIL-1ra treatment elicited paradoxically increased IL-1β and upregulated proteins associated with peripheral macrophage recruitment [121] such as monocyte chemoattractant protein-1 [125]. Further work must determine and extend the biological contextualization of these findings.

To summarize, robust clinical data demonstrate an injury-dependent altered neuroinflammatory response in the injured brain [17, 58, 108]. This response is CNS-specific and production of at least some core cytokines occurs in CNS compartments such as CSF and brain extracellular fluid [111]. As a core innate immune signaling pathway, the IL-1 system holds great promise as a therapeutic target. To date, few studies have investigated the clinical impact of IL-1β, IL-1α, and IL-1ra, but a high-quality evidence interventional study showed that the neuroinflammatory response following severe TBI is modulated following IL-1ra inhibition and that study drug administration was safe [21, 121]. Below, a synthesis of experimental and clinical findings and future research avenues are discussed.

## Discussion

Neuroinflammation is a core cellular injury mechanism following TBI. Inflammatory cells and mediators are likely to play a mechanistic role in development of secondary insults. We have summarized the strong evidence in favor of IL-1-modulation following experimental TBI, as well as initial promising clinical data in support of continued efforts in this domain. Below, we contextualize why we believe that pathophysiology-oriented neuroinflammatory modulation throughout interventional clinical trials is the next natural step in severe TBI research.

### rhIL-1ra Treatment Is Attainable, Safe, and Supported by Robust Experimental Work

We have reviewed the work that underlies our current knowledge of the IL-1 family, and these cytokines’ role following TBI. Within the experimental context, numerous studies demonstrate behavioral improvements that are paralleled by an altered neuroinflammatory response and other structural findings in rodents following TBI [83, 87–91, 95]. Of note, these results have emerged from different research groups across a long time period, thus speaking strongly in favor of reproducibility and thereby—a genuine treatment effect at the biological level. Yet, there are well-known differences between the rodent and human immune system [126]. Together, this raises the question as to whether the experimental biological effect is also clinically discernible.

In the clinical neurocritical care setting, observational data demonstrate increased protein levels of the IL-1 family following TBI [17]. A few clinical studies indicate that incremented levels of IL-1β are associated with poor prognosis [117, 118] and conversely that high levels of IL-1ra is neuroprotective [73]. Yet, this data was collected from small observational studies of mixed patient populations and should therefore be considered—at best—indicative of an association. Moreover, interventional trials of rhIL-1ra have inherent challenges. First, rhIL-1ra has a molecular mass of ~ 17 kDa [127], which has been hypothesized to limit CNS penetrance when administered peripherally [26, 74]. However, both peripheral intravenous [127] and subcutaneous administration [21] at a sufficient dose yield adequate CNS concentrations, making treatment feasible. Secondly, acute brain injury patients are notoriously difficult to include in randomized studies, as illustrated by Singh and colleagues, who administered rhIL-1ra to patients with aneurysmal subarachnoid hemorrhage in need of CSF drainage through a ventriculostomy. Their original power analysis suggested inclusion of *n* = 32 patients, whereas *n* = 13 were finally recruited. They found a small but non-significant reduction of IL-6 levels in the treated group [33]. This non-significant effect was likely because of the underpowered sample size, highlighting the high risk for type II errors in these patient segments.

In contrast, other non-traumatic brain injuries, characterized by an acute neuroinflammatory response [20], have been studied in interventional study designs. Here, a strong reduction in plasma IL-6 following rhIL-1ra treatment was seen following both aneurysmal subarachnoid hemorrhage [34] and also acute ischemic stroke [35]. Notably, across both studies, rhIL-1ra administration was proven safe [34, 35]. The latter is corroborated by the, to date, only randomized controlled trial of rhIL-1ra in severe TBI patients [21]. Importantly, it was shown that rhIL-1ra modified the neuroinflammatory response [121], the first study of its kind to demonstrate an actual biological treatment effect following severe TBI. The study was, however, not powered to assess outcome. Further phase III studies are therefore highly warranted, perhaps especially in the context of TBI, where no effective disease-modifying drug yet has been found [128].

### Neuroinflammatory Modulation Constitutes One Opportunity for Personalized TBI Treatment

The attempt to counteract TBI pathophysiology utilizing a biologically sound target constitutes a paradigm shift in TBI research. As rhIL-1ra is now proven to be safe while exerting an inflammation modifying effect [21, 121] speaks strongly in favor of both further studies utilizing the same mediator but—perhaps even more importantly—to elaborate in the field of neuroinflammation-targeted treatment following TBI. This is closely attached to the over-arching ambition of personalized treatment [3]. Paradoxically, in the absence of high-quality evidence, TBI is to some extent the diagnosis, in which treatment has always been individualized because of the inherent patient, injury, and secondary insult heterogeneity. In line with this, we suggest that future treatment should be directed towards pathophysiology-oriented treatment, of which neuroinflammation ought to be a core target that covers a range of possible mechanisms of injury [109].

To enable the eventual implementation of neuroinflammatory treatment, clinical routine warrants neuroinflammatory monitoring tools. Current TBI management is centered around multimodality-based approaches that ultimately strive to assess secondary insults [129]. Future advances require neuromonitoring to be directed also beyond secondary insults towards pathophysiology and cellular injury mechanisms. One tentative technique that is feasible for early implementation is fluid biomarkers that are readily available to quantify across both CSF and cerebral microdialysis. Numerous techniques are available and were recently reviewed [108]. This would naturally implement neuroinflammation in clinical decision making.

To enable the development of clinically beneficial treatments, outcome assessment tools likely need to be refined. Traditionally, the Glasgow Outcome Scale [130] has been utilized. This five-level ordinal scale stretching from dead to complete recovery was made more granular by the implementation of the extended Glasgow Outcome Scale [131]. Even though these scores encompass an overarching long-term functional assessment of patient status, they have been considered too crude [7], and the need for precise outcome metrics have been highlighted across international collaborative TBI efforts [7]. Within the clinical context, multi-dimensional outcome tools have been suggested [3]. Although these serve the purpose of a more complete outcome portrayal, pathophysiology-oriented treatment likely warrants pathophysiology-relevant outcome metrics. In the case of neuroinflammatory modulation, a secondary outcome (aside from safety, functional outcome) should likely be linked to the overarching neuroinflammatory response, as utilized by Helmy and colleagues [121]. As it is expectedly difficult to assess inter-dependent parallel processes with a common trigger, complex, multidimensional statistical techniques are likely warranted [110].

The work on rhIL-1ra should be viewed as the starting point for neuroinflammatory modulation following TBI, and we advise researchers to initiate additional interventional studies targeted towards neuroinflammation. As highlighted within a recent systematic review [116], different caspases constitute eligible targets. For example, caspase-1 is responsible for the cleavage of pro-IL-1β into its mature form [13]; this aligns with the overall benefit of neuroinflammatory modulation. Other suggested treatment targets are IL-6 [117, 118] and complement [58, 109]. The latter is in fact currently initiated as an ongoing trial [71]. Importantly, this review puts a clear focus in the domain of acute innate neuroinflammation, whereas there is a growing interest also in adaptive responses. Among else, the long-term development of autoantibodies [132] seems to be of importance, thus speaking in favor of continued neuroinflammatory vigilance following the acute trauma phase.

To summarize, TBI research holds the opportunity of entering a new era of pathophysiology-oriented treatment. Neuroinflammatory-focused treatment is feasible, as demonstrated above, even though the tentative clinical benefit remains to be demonstrated in the clinical context.

## Conclusion

Neuroinflammatory modulation following severe TBI is biologically rational, as proven in a rich amount of experimental studies. However, to this point, only one interventional neuroinflammatory-modulating trial has been undertaken following severe TBI. Aside from showing clinical safety and feasibility, this treatment also demonstrates that the neuroinflammatory response can be modulated following severe TBI, thus initiating a new era of pathophysiology-oriented treatment. Future experimental and clinical studies specifically addressing target-defined facets of secondary injury are warranted.

## Abbreviations

ASC: Apoptosis-associated speck-like protein containing a caspase recruiting domain
BBB: Blood-brain barrier
BDNF: BRAIN-derived neurotrophic factor
CENTER-TBI: Collaborative European NeuroTrauma Effectiveness Research study
CNS: Central nervous system
CSF: Cerebrospinal fluid
DAMP: Damage-associated molecular pattern
HMGB1: High-mobility group box 1
IL-: Interleukin-
IL-1ra: IL-1 receptor antagonist
IL-1R1: Type I IL-1 receptor
IL-1R2: Type II IL-1 receptor
(NFκB): Nuclear factor kappa light-chain-enhancer of activated B cells
NLR: Nucleotide-binding oligomerization domain-like receptor
NLRP: NLR family pyrin domain containing (NLRP)
PRR: Pattern recognition receptor
rh: Recombinant human
TBI: Traumatic brain injury
TLR: Toll-like receptor
TRACK-TBI: Transforming Research and Clinical Knowledge in TBI
TNF: Tumor necrosis factor
